# Clinical characteristics and factors associated with psychiatric comorbidity among people with epilepsy in Ganzi Tibetan autonomous prefecture of China: a cross-sectional study

**DOI:** 10.1186/s42494-026-00256-4

**Published:** 2026-04-07

**Authors:** Chunxia Wang, Deng Chen, Liusha Zhao, Fangzhou Liu, Ling Zhang, Xiaoyan Liu, Ling Liu

**Affiliations:** 1 Department of Neurology, West China Hospital, Sichuan University, Chengdu, Sichuan 610041 China; 2 Department of Neurology, 363 Hospital, Chengdu, Sichuan 610041 China; 3 Chengdu Eighth People’s Hospital (Geriatric Hospital of Chengdu Medical College), Chengdu, Sichuan 610041 China; 4 Department of Neurology, The First People’s Hospital of Longquanyi District, Chengdu, Sichuan 610041 China

**Keywords:** Epilepsy, Depression, Anxiety, Quality of life, Tibetan area, Cross-sectional study, Prevalence

## Abstract

**Background:**

This study was conducted to determine the prevalence of epilepsy in Daofu County, Sichuan Province, China, and to identify factors associated with comorbid depression, anxiety, and impaired quality of life among people with epilepsy (PWE).

**Methods:**

In this community-based, cross-sectional study, a door-to-door survey of epilepsy was performed among 37,390 permanent residents of Daofu County. The survey identified 164 confirmed cases of epilepsy. Of them, 127 participants (aged ≥ 16 years) who were able to complete self-reported questionnaires were enrolled for final analysis. Depression and anxiety symptoms were assessed using the Chinese versions of the Neurological Disorders Depression Inventory for Epilepsy (C-NDDI-E) and the Generalized Anxiety Disorder-7 (GAD-7) scale, respectively. Health-related quality of life was evaluated with the Quality of Life in Epilepsy Inventory-31 (QOLIE-31). Factors associated with depression and anxiety were examined using logistic regression analyses, while factors influencing QOLIE-31 scores were assessed by linear regression.

**Results:**

The estimated prevalence of epilepsy in Daofu County was 4.4‰ (95% CI: 3.7–5.1‰). Independent factors associated with the comorbid depression included a low monthly household income per capita (aOR = 4.41, 95% CI: 1.82–10.71; *P* = 0.001) and active epilepsy (aOR = 4.87, 95% CI: 1.74–13.62; *P* = 0.003). Independent factors associated with the comorbid anxiety included a low monthly household income per capita (aOR = 5.03, 95% CI: 1.88–13.5; *P* = 0.001) and active epilepsy (aOR = 4.84, 95% CI: 1.95–12.01; *P* = 0.001). Furthermore, factors including a low monthly household income per capita, a history of status epilepticus, and active epilepsy were significantly associated with a lower QOLIE-31 score.

**Conclusions:**

There is a high burden of depression and anxiety among PWE in the Tibetan areas of China. Active epilepsy is the most potent, independent risk factor of psychiatric comorbidities and poorer quality of life. These findings suggest the urgent need for integrated epilepsy management and mental health services in this resource-limited region.

## Background

Epilepsy is one of the most prevalent chronic neurological disorders worldwide, affecting approximately 70 million people globally [1], and over 80% of them reside in low- and middle-income countries [2]. Epilepsy is frequently complicated by a range of neuropsychiatric comorbidities, notably depression and anxiety [3]. The lifetime prevalence of these comorbidities among people with epilepsy (PWE) is reported to be 30–50%, substantially higher than that in the general population [4]. These psychiatric comorbidities are closely linked to poor seizure control, suboptimal treatment adherence, and poor quality of life, constituting the core clinical challenge from the bidirectional relationship between epilepsy and psychiatric disorders [5].

In China, epilepsy imposes a substantial public health burden. Although national epidemiological data suggest a prevalence of 4–7‰, significant regional disparities exist [6, 7]. In regions with limited healthcare resources, the rates of diagnosis, treatment, and management of comorbidities among PWE are likely even lower due to various factors such as geographic location, socioeconomic status, and ethnicity [8]. Sitting on the eastern edge of the Qinghai-Xizang Plateau and at an altitude of over 3500 m, Ganzi Tibetan autonomous prefecture in Sichuan Province is characterized by vast territory, and low population density. The limited medical resources in this region pose a substantial barrier to the accessibility to standardized epilepsy care. Daofu County, in Ganzi prefecture, with a total population of 52,200, is facing such challenge. Nevertheless, systematic data on the epidemiological characteristics, clinical profiles, and patterns of psychiatric comorbidity among PWE in this region remain scarce. This knowledge gap severely hinders the development and targeted implementation of effective public health interventions for PWE.

In this study, we performed a survey on the prevalence of epilepsy in Daofu County. The aim of this study was to explore differences in sociodemographic and clinical characteristics between patients with active and inactive epilepsy, and analyze factors associated with psychiatric comorbidities and quality of life of PWE in this Tibetan area.

## Materials and methods

### Study design and study population

This community-based, cross-sectional study was conducted in Daofu County, Ganzi Tibetan autonomous prefecture, Sichuan Province, between June and October in 2025. The study was performed in accordance with the Declaration of Helsinki and approved by the Biomedical Ethics Review Committee of West China Hospital, Sichuan University (Approval No. 2025–2092). Written informed consent was obtained from all participants or their legal guardians before enrollment. The survey encompassed all 19 administrative villages within the county. In collaboration with the Daofu County Health Bureau and its subordinate township health centers, we obtained a comprehensive roster of all permanent residents. The permanent residents were defined as individuals officially registered as permanent residents of the county during the survey period, with temporary absences not exceeding one month. A final sample of 37,390 individuals was thus established.

### Epidemiological survey

The survey was conducted in three phases. In Phase I, locally recruited healthcare workers conducted door-to-door screening of all eligible residents, using a questionnaire developed based on a standard screening tool recommended by the World Health Organization [9]. The detailed questionnaire items are provided in Appendix A. Respondents aged ≥ 14 years (with normal intellectual ability) underwent direct interviews; children under 14 years and individuals with intellectual disabilities were surveyed by interviewing their parents or guardians. Participants answering “yes” to any item were classified as suspected cases of epilepsy. All participating healthcare personnel underwent a two-day standardized training led by neurologists from West China Hospital. The epilepsy screening questionnaire was translated into Tibetan language and back-translated to ensure linguistic accuracy. Inter-rater reliability assessed in a 50-person pilot sample yielded a Kappa coefficient of 0.85 (95% confidence interval: 0.72–0.94).

In Phase II, all suspected cases identified during initial screening were directly reviewed (face-to-face) by experienced neurologists to confirm or rule out the diagnosis; for difficult-to-diagnose cases, discussions were made with neurological specialists at West China Hospital. For confirmed cases, sociodemographic data collected, including age, sex, occupation, education level, per capita monthly household income, and place of residence. Diagnosis of epilepsy, etiology, history of status epilepticus(SE), and baseline seizure frequency were obtained by reviewing medical records and interviews with the patients or their caregivers. The date of initial presentation, disease duration, classification as active or inactive epilepsy, and current treatment status were determined based on patient self-reports and clinical assessments by neurologists at the time of survey. Past neurological diagnostic evaluations, such as brain imaging (computed tomography [CT] or magnetic resonance imaging [MRI]) and electroencephalography (EEG or video EEG), were also systematically documented when available.

Phase III was dedicated to the specialized assessment of neuropsychiatric comorbidities, using strict inclusion and exclusion criteria. The inclusion criteria were: (1) age 16 years or older; and (2) provision of written informed consent by the participant or their legal guardian. The exclusion criteria comprised conditions that may preclude reliable completion of psychological assessments, including moderate-to-severe intellectual disability, schizophrenia, or other major psychiatric disorders. Participants meeting the inclusion criteria underwent assessments for anxiety (GAD-7 scale), depression (C-NDDI-E scale), and quality of life (QOLIE-31 scale).

### Epilepsy screening and diagnosis

The diagnosis of epilepsy was based on the 2014 International League Against Epilepsy (ILAE) criteria: (1) at least two unprovoked seizures occurring over 24 h apart; or (2) a single unprovoked seizure with a high probability of recurrence based on EEG showing epileptiform discharges or neuroimaging revealing lesions associated with a persistent seizure tendency. Alternatively, in the absence of these tests, a detailed clinical history indicating high recurrence risk (such as focal seizure symptoms, nocturnal seizures, family history of epilepsy, or abnormal neurological examination) may be used [10].

SE was defined according to the 2015 ILAE criteria as follows: (1) a continuous seizure lasting > 5 min; or (2) two or more discrete seizures without full recovery of consciousness between them [11].

Following the ILAE 2017 framework and considering the practical constraints of this resource-limited setting, epilepsy etiology was categorized into two types: (1) epilepsy of unknown etiology, where no identifiable cause was found; and (2) epilepsy of known etiology, attributed to identifiable structural damage or infection in brain [10, 12].

Active epilepsy was identified as patient havings experienced two or more spontaneous seizures on different dates within the past year [13].

The treatment status was categorized into three types: untreated, standard treatment, and non-standard treatment. Standard treatment refers to sustained treatment with an antiseizure medication according to standardized protocols. Non-standard treatment includes traditional Chinese medicine, folk remedies, and inappropriate treatment methods [14, 15].

Baseline seizure frequency was defined as the mean monthly number of seizures during the 12 months before initiation of the current antiseizure medication (ASM) regimen. For treatment‑naïve patients, the baseline seizure frequency was defined as the mean monthly seizure count over 12 months before the survey. The frequency was categorized as low (< 1 seizure per year), moderate (≥ 1 per year but ≤ 1 per month), or high (> 1 per month).

### Questionnaires

The Tibetan versions of C-NDDI-E, GAD-7, and QOLIE-31 were used. The Chinese versions were translated into Tibetan by a local bilingual physician (native Tibetan speaker) and independently back-translated into Chinese to ensure conceptual equivalence; discrepancies were resolved through discussion. For the 43 illiterate participants (33.9%), questionnaires were administered orally by trained local Tibetan-speaking healthcare workers. To minimize interviewer bias, all questions were read verbatim without rephrasing, and responses were transcribed exactly.

#### Depression assessment

Depressive symptoms were assessed using the C-NDDI-E. The C-NDDI-E is a widely used depression screening tool for PWE with good reliability and validity. A score > 12 indicates depressive comorbidity [16].

#### Anxiety assessment

Anxiety was assessed using the Chinese version of GAD-7. According to the validation study of this scale in Chinese PWE, a cutoff score of > 6 was used to define the comorbid anxiety [17].

#### Quality of life assessment

Quality of life was assessed using the Chinese version of QOLIE-31. This 31-item scale covers seven domains: seizure-related concerns, overall quality of life, emotional health, energy level, cognitive function, medication side effects, and social functioning. Domain scores were transformed linearly to a 0–100 scale, with higher scores indicating better quality of life. The overall score was calculated as a weighted average of the domain scores according to the instrument's scoring manual [18]. No participants missed the data that precluded calculation of the overall QOLIE-31 score.

### Statistical analysis

All 127 participants completed the C-NDDI-E, GAD-7, and QOLIE-31 questionnaires without missing items. Descriptive statistics were computed for all study participants. Categorical variables are summarized as frequencies and percentages, and continuous variables as mean ± standard deviation (SD). For group comparisons, one-way analysis of variance (ANOVA) or the Kruskal–Wallis test was performed for continuous variables, and the chi-square test or Fisher’s exact test for categorical variables. Univariate logistic regression was first performed to identify potential factors associated with depression and anxiety. Variables with a significance level of *P* < 0.05 in the univariate analysis were further analyzed using a multivariable logistic regression model with a stepwise selection procedure. Factors associated with quality of life (QOLIE-31 scores) were analyzed using the multivariable linear regression method. All analyses were performed using R software (version 4.3.0; R Foundation for Statistical Computing) and Free Statistics software (version 2.2, https://www.clinicalscientists.cn, Beijing). A two-tailed *P* value < 0.05 was considered statistically significant.

## Results

### Epilepsy prevalence and patient characteristics

In this study, door-to-door screening of 37,390 permanent residents in Daofu County identified 164 confirmed cases of epilepsy, yielding a crude prevalence rate of 4.4‰ (164/37,390, 95% CI: 3.7–5.1‰). Among these patients, 144 (87.8%) had sought medical consultation at tertiary hospitals. In terms of diagnostic workup, 136 patients (82.9%) had received either CT or MRI, and 128 patients (78.0%) had received EEG or video-EEG monitoring.

Regarding etiological composition, the top three identified causative factors were intracranial parasitic infections (*n* = 27, 16.5%), traumatic brain injury (TBI) (*n* = 18, 11.0%), and post-stroke epilepsy (*n* = 12, 7.3%). The etiology remained unknown for 53 patients (32.3%). Other identified causes included unexplained intracranial space-occupying lesions, brain tumors, and less common conditions such as cortical dysplasia and hippocampal sclerosis. In terms of treatment status, 137 patients (83.5%) were on one or more anti-seizure medications. Additionally, 51 patients (31.1%) had a history of or were currently receiving traditional Tibetan medicine therapy.

**Fig. 1:**
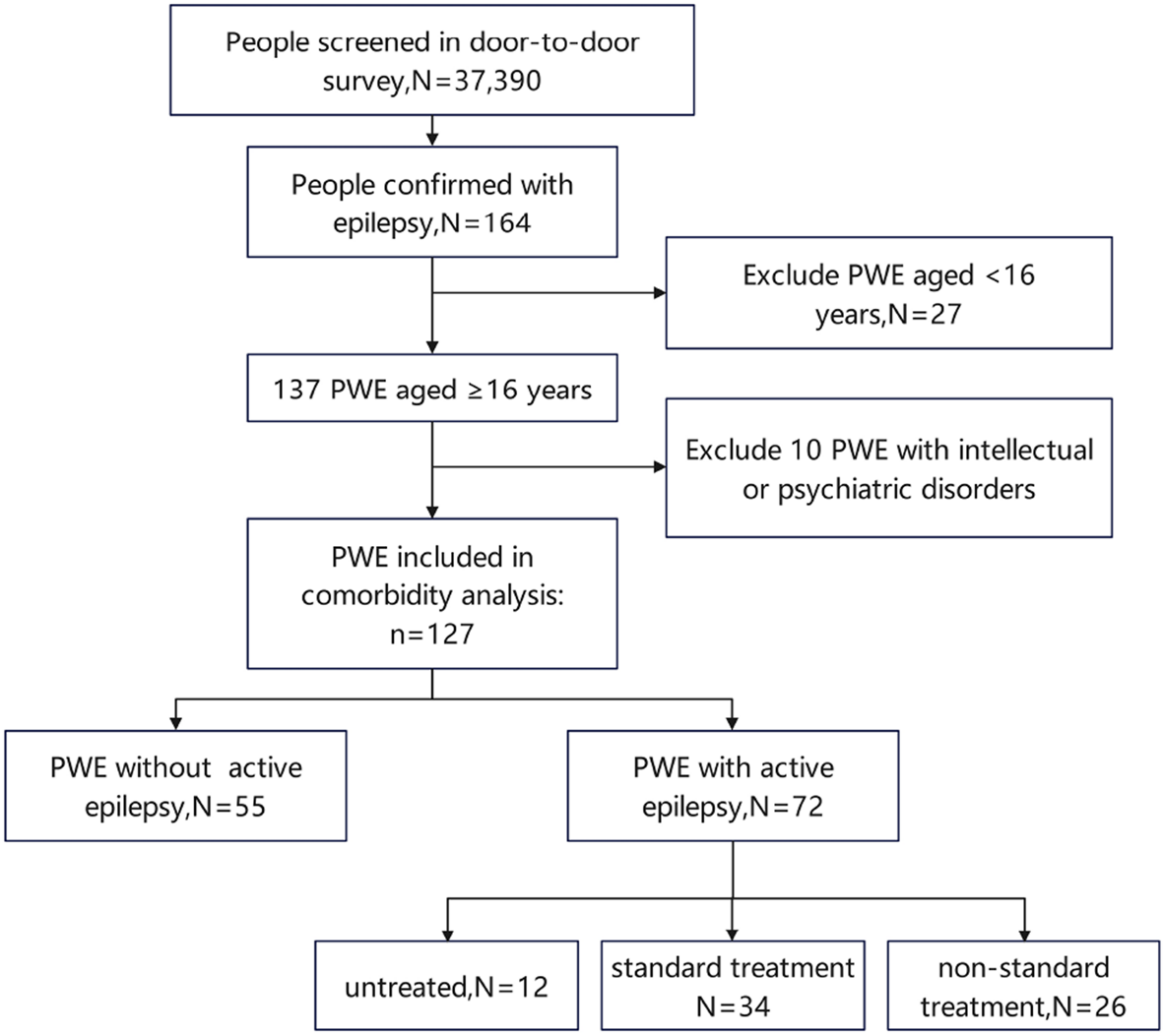
Flow chart of participant inclusion

Further, 27 patients aged < 16 years and 10 patients unable to complete assessments due to severe mental disorders or intellectual disabilities, were excluded from comorbidity analysis (Fig. 1), to ensure eligibility of self-reported comorbidities. Among the resulting 127 PWE, the prevalence of depression and anxiety were 35.4% and 52.0%, respectively. The cohort of 127 PWE had a mean age of 41.6 ± 16.6 years, including 77 males (60.6%) and 50 females (39.4%). The patients were categorized into inactive (*n* = 55, 43.3%) and active epilepsy (*n* = 72, 56.7%) groups. Compared to the inactive group, patients with active epilepsy were significantly younger (38.3 ± 14.5 vs. 45.8 ± 18.2 years, *P* = 0.011), and more likely to have a monthly household income < 1000 CNY (50.0% vs. 14.5%, *P* < 0.001), a history of SE (38.9% vs. 14.5%, *P* = 0.003) and a high baseline seizure frequency (55.6% vs. 20.0% for ≥ 1/month frequency, *P* < 0.001). The detailed sociodemographic and clinical characteristics are presented in Table 1.

**Table 1:** Demographics and clinical characteristics of PWE (active versus non-active epilepsy)

Variables	Total (*n* = 127)	Non-active epilepsy (*n* = 55)	Active epilepsy (*n* = 72)	*P-*value
Sex, *n* (%)				0.811
Female	50 (39.4)	21 (38.2)	29 (40.3)	
Male	77 (60.6)	34 (61.8)	43 (59.7)	
Age (years), Mean ± SD	41.6 ± 16.6	45.8 ± 18.2	38.3 ± 14.5	0.011*
Occupation, *n* (%)				0.637
Not employed	15 (11.8)	6 (10.9)	9 (12.5)	
Farmer	70 (55.1)	29 (52.7)	41 (56.9)	
Employee	28 (22.0)	15 (27.3)	13 (18.1)	
Student	14 (11.0)	5 (9.1)	9 (12.5)	
Education status, *n* (%)				0.515
Illiterate	43 (33.9)	18 (32.7)	25 (34.7)	
Elementary school	33 (26.0)	17 (30.9)	16 (22.2)	
Junior high school	34 (26.8)	15 (27.3)	19 (26.4)	
College	17 (13.4)	5 (9.1)	12 (16.7)	
Monthly household income per capita, *n* (%)				< 0.001*
≥ 1000 CNY	83 (65.4)	47 (85.5)	36 (50)	
< 1000 CNY	44 (34.6)	8 (14.5)	36 (50)	
Living area, *n* (%)				0.634
Rural/pastoral	86 (67.7)	36 (65.5)	50 (69.4)	
Urban	41 (32.3)	19 (34.5)	22 (30.6)	
Convulsive seizures, *n* (%)				0.097
No	26 (20.5)	15 (27.3)	11 (15.3)	
Yes	101 (79.5)	40 (72.7)	61 (84.7)	
Etiology,* n* (%)				0.542
Epilepsy of unknown etiology	47 (37.0)	22 (40)	25 (34.7)	
Epilepsy of known etiology	80 (63.0)	33 (60)	47 (65.3)	
History of status epilepticus, *n* (%)				0.003*
No	91 (71.7)	47 (85.5)	44 (61.1)	
Yes	36 (28.3)	8 (14.5)	28 (38.9)	
Treatment, *n* (%)				0.34
Untreated	18 (14.2)	6 (10.9)	12 (16.7)	
Regular treatment	67 (52.8)	33 (60)	34 (47.2)	
Irregular treatment	42 (33.1)	16 (29.1)	26 (36.1)	
Time to first medical contact after seizure onset, *n* (%)				0.818
> 12 months	36 (28.3)	14 (25.5)	22 (30.6)	
1–12 months	33 (26.0)	15 (27.3)	18 (25)	
< 1 month	58 (45.7)	26 (47.3)	32 (44.4)	
Disease duration (years), *n* (%)				0.66
1–4	40 (31.5)	17 (30.9)	23 (31.9)	
5–9	25 (19.7)	13 (23.6)	12 (16.7)	
10–19	31 (24.4)	11 (20)	20 (27.8)	
≥ 20	31 (24.4)	14 (25.5)	17 (23.6)	
Baseline seizure frequency, *n* (%)				< 0.001*
low	16 (12.6)	12 (21.8)	4 (5.6)	
moderate	60 (47.2)	32 (58.2)	28 (38.9)	
high	51 (40.2)	11 (20)	40 (55.6)	

Data were analyzed with Chi-square test, Fisher's exact test, or independent *t*-test, as appropriate. Monthly household income per capita was defined as the total monthly household income (in Chinese Yuan) divided by the number of household members, based on self-report. The 1000 CNY was selected as the cut-off based on the local poverty line in Ganzi prefecture at the time of survey (2025). All income data refer to nominal values for the year 2025

^*^P < 0.05

### Analysis of factors influencing the depression comorbidity

Of the 127 PWE, 45 (35.4%; 95% CI: 27.1–43.8%) met the criteria for comorbid depression. Univariate logistic regression (Table 2) showed that older age (*P* = 0.047), lower monthly household income (*P* < 0.001), higher baseline seizure frequency (*P* = 0.039), and active epilepsy (*P* < 0.001) were significantly associated with depression. After multivariable adjustment, the factors of monthly household income < 1000 CNY (aOR = 4.41, 95% CI: 1.82–10.71; *P* = 0.001) and active epilepsy (aOR = 4.87, 95% CI: 1.74–13.62; *P* = 0.003) remained independently associated with the depressive comorbidity (Table 4).

**Table 2:** Univariate logistic regression analysis of factors associated with depression in PWE

Variables	OR (95% CI)	*P-*value
Sex		
Female	Ref.	
Male	1.11 (0.53–2.34)	0.786
Age, years	0.98 (0.95–1)	0.047*
Occupation		
Not employed	Ref.	
Farmer	0.94 (0.3–2.94)	0.918
Employee	0.5 (0.13–1.91)	0.311
Student	0.83 (0.19–3.75)	0.812
Education status		
Illiterate	Ref.	
Elementary school	0.76 (0.3–1.97)	0.579
Junior high school	1.07 (0.43–2.68)	0.884
College	0.33 (0.08–1.31)	0.115
Monthly household income per capita		
≥ 1000 CNY	Ref.	
< 1000 CNY	6.79 (3.01–15.32)	< 0.001*
Living area		
Rural/pastoral	Ref.	
Urban	0.66 (0.3–1.48)	0.317
Convulsive seizures		
No	Ref.	
Yes	2.75 (0.96–7.9)	0.06
Etiology		
Epilepsy of unknown etiology	Ref.	
Epilepsy of known etiology	0.71 (0.34–1.5)	0.368
History of status epilepticus		
No	Ref.	
Yes	2.01 (0.91–4.44)	0.083
Treatment Status		
Untreated	Ref.	
Standard treatment	0.37 (0.13–1.07)	0.067
Non-standard treatment	0.75 (0.25–2.27)	0.611
Time to first medical contact after seizure onset		
> 12 months	Ref.	
1–12 months	1.02 (0.39–2.69)	0.966
< 1 month	0.71 (0.3–1.69)	0.435
Disease duration (years)		
1–4	Ref.	
5–9	0.46 (0.14–1.5)	0.201
10–19	1.53 (0.59–4)	0.386
≥ 20	1.17 (0.44–3.1)	0.748
Baseline seizure frequency		
Low	Ref.	
Moderate	3.77 (0.78–18.19)	0.098
High	5.31 (1.09–25.83)	0.039*
Active epilepsy		
No	Ref.	
Yes	7.66 (3.06–19.19)	< 0.001*

*Abbreviations: OR* odds ratio, *CI* confidence interval, *Ref* reference category

Each odds ratio represents the unadjusted association between the specified variable and depression

Depression was defined as a score > 12 on the Chinese version of the Neurological Disorders Depression Inventory for Epilepsy (C-NDDI-E)

### Analysis of factors influencing the anxiety comorbidity

Of the 127 PWE, 66 (52.0%; 95% CI: 43.3–60.7%) met the criteria for comorbid anxiety. Univariate analysis (Table 3) showed that low household income (*P* < 0.001), a history of SE (*P* = 0.005), high baseline seizure frequency (*P* = 0.004), and active epilepsy (*P* < 0.001) were significantly associated with anxiety. After multivariable adjustment, monthly household income < 1000 CNY (aOR = 5.03, 95% CI: 1.88–13.5; *P* = 0.001) and active epilepsy (aOR = 4.84, 95% CI: 1.95–12.01; *P* = 0.001) remained independently associated with the anxiety comorbidity (Table 4).

**Table 3:** Univariate logistic regression analysis of factors associated with anxiety in PWE

Variables	OR (95% CI)	*P-*value
Sex		
Female	Ref.	
Male	1 (0.49–2.03)	0.995
Age, years	0.99 (0.97–1.01)	0.431
Occupation		
Not employed	Ref.	
Farmer	0.67 (0.21–2.07)	0.484
Employee	0.58 (0.16–2.06)	0.398
Student	1.2 (0.27–5.4)	0.812
Education status		
Illiterate	Ref.	
Elementary school	1.38 (0.56–3.43)	0.488
Junior high school	1.86 (0.74–4.64)	0.185
College	0.81 (0.26–2.51)	0.708
Monthly household income per capita		
≥ 1000 CNY	Ref.	
< 1000 CNY	7.95 (3.27–19.31)	< 0.001*
Living area		
Rural/pastoral	Ref.	
Urban	0.72 (0.34–1.51)	0.382
Convulsive seizures		
No	Ref.	
Yes	1.34 (0.56–3.18)	0.507
Etiological Classification		
Epilepsy of unknown etiology	Ref.	
Epilepsy of known etiology	0.81 (0.39–1.66)	0.563
History of status epilepticus		
No	Ref.	
Yes	3.31 (1.43–7.67)	0.005*
Treatment Status		
Untreated	Ref.	
Standard treatment	0.91 (0.32–2.59)	0.866
Non-standard treatment	1.47 (0.48–4.46)	0.496
Time to first medical contact after seizure onset		
> 12 months	Ref.	
1–12 months	1.2 (0.47–3.09)	0.706
< 1 month	1.07 (0.47–2.46)	0.871
Disease course (years)		
1–4	Ref.	
5–9	0.39 (0.14–1.1)	0.074
10–19	1.13 (0.44–2.92)	0.796
≥ 20	1.13 (0.44–2.92)	0.796
Baseline seizure frequency		
Low	Ref.	
Moderate	2.45 (0.71–8.49)	0.156
High	6.56 (1.83–23.53)	0.004*
Active epilepsy		
No	Ref.	
Yes	9.01 (4–20.33)	< 0.001*

*Abbreviations*: *OR* odds ratio, *CI* confidence interval, *Ref* reference category

Each odds ratio represents the unadjusted association between the specified variable and anxiety

Anxiety was defined as a score > 6 on the Generalized Anxiety Disorder-7 (GAD-7) scale, based on the validation study in Chinese people with epilepsy

**Table 4:** Multivariable regression analysis of factors associated with depression, anxiety, and quality of life in PWE

	**Depression**		**Anxiety**		**QOLIE-31 score**	
**Variable**	**adj. OR (95% CI)**	**adj. *P-*value**	**adj. OR (95% CI)**	**adj. *P-*value**	**adj. Coefficient (95% CI)**	**adj. *P-*value**
Monthly household income per capita						
≥ 1000 CNY	1 (Ref)		1 (Ref)		0 (Ref)	
< 1000 CNY	4.41 (1.82 −10.71)	0.001*	5.03 (1.88 −13.5)	0.001*	−13.4 (−18.19 to −8)	< 0.001*
History of status epilepticus						
No					0 (Ref)	
Yes					−8.69 (−13.57 to −3.8)	0.001*
Active epilepsy						
No	1 (Ref)		1 (Ref)		0 (Ref)	
Yes	4.87 (1.74 −13.62)	0.003*	4.84 (1.95 −12.01)	0.001*	−24.54 (−29.28 to −19.8)	< 0.001*

^*^Abbreviations: *adj. OR* adjusted odds ratio, *CI* confidence interval, *QOLIE-31* 31-item Quality of Life in Epilepsy Inventory *

^*^Variables shown are those remaining in the final multivariable regression model. The reference group (Ref) for each categorical variable is indicated. Coefficient estimates are presented for the QOLIE-31 total score linear regression model *

### Analysis of factors affecting quality of life

Univariate linear regression analysis showed that older age (*P* = 0.027), lower household income (*P* < 0.001), a history of SE (*P* < 0.001), higher baseline seizure frequency (*P* < 0.001), and active epilepsy (*P* < 0.001) were significantly associated with lower QOLIE-31 scores. After multivariable adjustment, monthly household income < 1000 CNY (β = −13.44, 95% CI: −18.19 to −8.0; *P* < 0.001), a history of SE (β = −8.69, 95% CI: −13.57 to −3.8; *P* = 0.001), and active epilepsy (β = −24.54, 95% CI: −29.28 to −19.8; *P* < 0.001) remained independently associated with poorer quality of life (Table 4).

A post-hoc variance inflation factor (VIF) analysis was performed to verify collinearity for all key variables (active epilepsy, history of SE, baseline seizure frequency, and monthly household per capita income) included in the multivariate logistic (depression/anxiety) and linear (QOLIE-31) regression model. All variables had VIF values ranging 1.12–1.86 (all < 2), well below the critical threshold of 10. This confirmed the absence of significant multicollinearity among these variables and validated the independent predictive value of variables identified in each regression model.

### Quality of life radar chart analysis

**Fig. 2:**
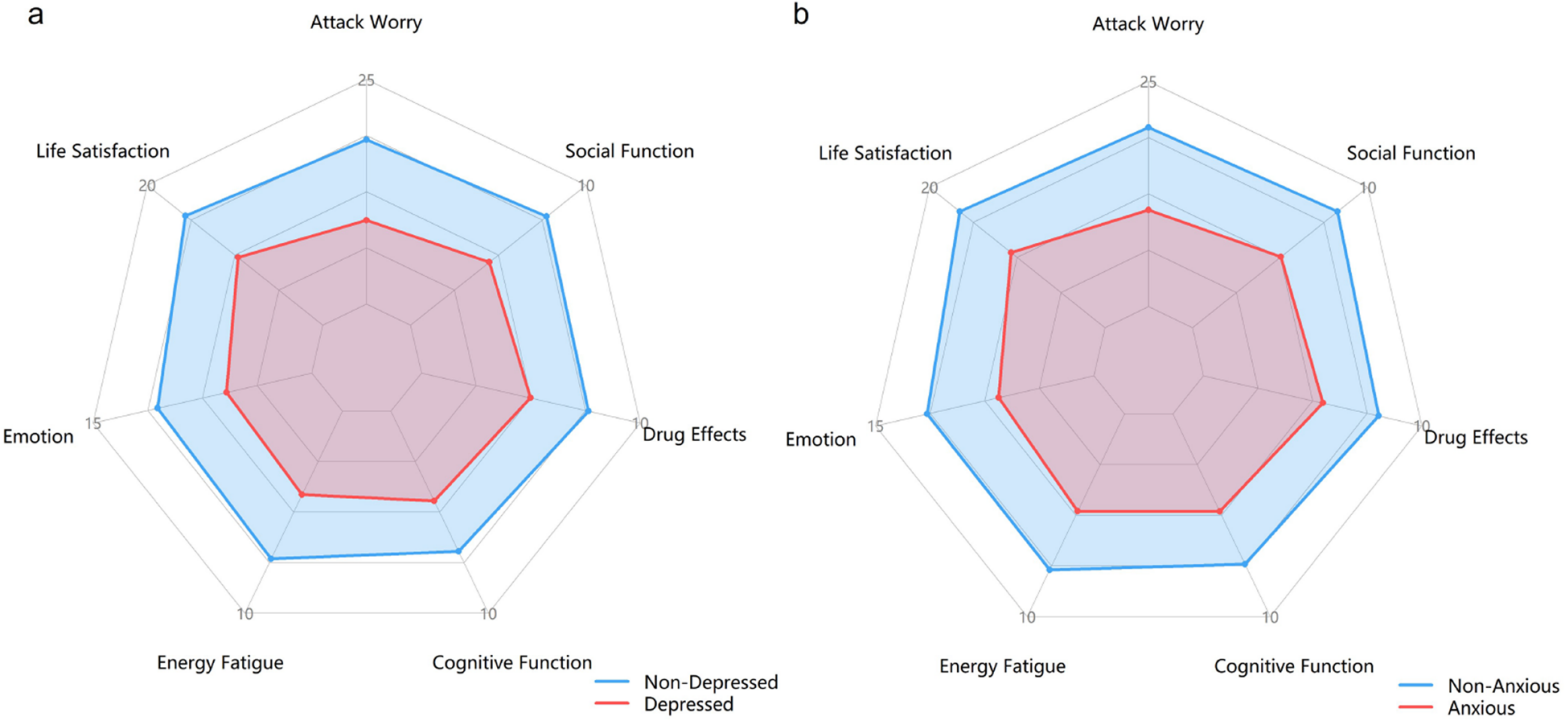
**a** Radar plot of PWE with versus without depression. **b** Radar plot of PWE with vs. without anxiety

The mean scores of QOLIE-31 domains were compared between patients with and without depression and between patients with and without anxiety, as shown in the radar charts. Compared to those without depression, PWE with depression had lower mean scores across most domains, with notably lower scores in emotional well-being and energy (Fig. 2a). Similarly, PWE with anxiety showed lower mean scores than those without anxiety, particularly evident in emotional well-being and seizure worry (Fig. 2b).

## Discussion

### Epilepsy prevalence and etiological features in the tibetan area

This study was the first systematic investigation of the prevalence and psychiatric comorbidities of epilepsy in the Tibetan community in Ganzi prefecture. The survey was conducted in Daofu County, a region situated on the eastern edge of the Qinghai-Xizang Plateau at an average altitude of approximately 3,500 m, characterized by high mountains and valleys. As an economically underdeveloped area, the county's economy relies primarily on agriculture and animal husbandry. The annual gross domestic product (GDP) is approximately 2 billion CNY, with an average per capita income of ~ 15,000 CNY among farmers and herdsmen. Over 90% of the population is of Tibetan ethnicity [19].

The estimated epilepsy prevalence in Daofu County was 4.4‰, consistent with reports from other regions of China [6, 20]. Notably, a substantial proportion of patients had sought care at tertiary hospitals, and the proportion of patients receiving neuroimaging (e.g., CT/MRI) and EEG was considerably higher than those documented in previous studies [8, 21]. This improvement may reflect a gradual improvement of local transportation infrastructure, increased public and patient awareness of epilepsy, more proactive healthcare-seeking behavior, and sustained socioeconomic progress. Collectively, these developments led to improved accessibility and utilization of standardized diagnostic and treatment services for epilepsy in this region.

Regarding etiological composition, parasitic infections (*n* = 27, 16.5%) and TBI (*n* = 18, 11.0%) were the leading causes, consistent with a previous study in Ganzi Prefecture that reported a similar proportion (14.6%) for parasitic infections [21]. Parasitic infection remains a significant public health concern in this region [22], closely linked to local dietary practices—particularly consumption of raw or undercooked pork and beef—and suboptimal sanitation conditions. Targeted prevention and control strategies for parasitic infection are therefore warranted while carefully balancing the preservation of local cultural practices.

### High burden of psychiatric comorbidities in the study cohort

The prevalence of depression (35.4%) and anxiety (52.0%) in our study was markedly higher than those reported in a national multicenter study (depression: 27.3%; anxiety: 31.5%) and a western China survey (depression: 29.9%; anxiety: 33.4%) in Chinese PWE [23–25]. The increased prevalence indicates elevated comorbidity burden, which aligns with observations from rural, resource-limited settings in low- and middle-income countries (LMICs). A systematic review of LMICs reported prevalence ranging 24.4–89.9% for depression and 1.9–84.2% for anxiety [3]. Similar findings have been documented in Morocco (depression: 44.9%; anxiety: 51.4%) and Ethiopia (pooled estimates: depression 37.1%; anxiety 33.8%) [26, 27]. The consistent observation of high comorbidity rates across geographically diverse but socioeconomically comparable settings suggests that the limited healthcare access, inadequate seizure control, financial burden, and social stigma collectively create an environment that elevates the psychiatric risk. Thus, the mental health burden identified in Daofu County reflects a broader global challenge among PWE in resource-constrained settings. These findings underscore the urgent need to integrate mental health screening and intervention into routine epilepsy care and to develop targeted, community-based supporting programs.

### Socioeconomic status as a critical determinant of psychiatric comorbidity

In this study, a monthly household income below 1000 CNY was reported by 62.2% (28/45) of epilepsy patients with depression and 54.5% (36/66) of those with anxiety, consistent with a systematic review linking lower socioeconomic status to a higher depression prevalence [24]. Multivariate analysis confirmed that the low-income patients were facing markedly elevated risks: a nearly fourfold increase for depression (aOR = 4.41) and a fivefold increase for anxiety (aOR = 5.03). This can be explained from two perspectives: low income limits access to continuous, qualified healthcare, and on the other hand amplifies daily psychological stress and social burdens such as stigma [28]. Conversely, the psychiatric comorbidities can further exacerbate the economic status due to impaired work capacity, additional mental healthcare costs, and increased exposure to stigma-related social exclusion [29]. These financial constraints may initiate a vicious cycle, whereby the inability to afford long-term ASM therapy leads to suboptimal seizure control, which in turn worsens both psychiatric and socioeconomic conditions.

### Active epilepsy as the strongest risk factor

Patients with active epilepsy have significantly poorer psychosocial outcomes compared to those with well-controlled seizures [30]. In this study, active epilepsy was found to be the strongest independent risk factor, associated with a nearly fivefold increase in depression (aOR = 4.87) and anxiety (aOR = 4.84), and a 24.5-point reduction in QOLIE-31 score. Inadequate seizure control may exert dual detrimental effects: impairing the neurological functions and indirectly increasing the susceptibility to psychiatric comorbidity [5]. These findings support the hypothesis that epilepsy shares common neurobiological underpinnings with certain neuropsychiatric disorders. Previous studies have identified several potential mechanisms, including shared genetic susceptibility, disturbances in neurotransmitter systems, structural or functional abnormalities in specific brain regions (such as limbic circuits), dysfunction of the hypothalamic–pituitary–adrenal (HPA) axis, and inflammatory processes [31]. These shared pathophysiological pathways may contribute to the well-established bidirectional relationship between epilepsy and psychiatric disorders.

In this area inhabited by the Tibetan ethnic group, uncontrolled seizures directly increase the vulnerability to major psychosocial stress, including unemployment, social isolation, and disrupted interpersonal relationships [30]. This psychosocial stress, combined with the stress from the disease per se, compounds the overall psychological burden. Furthermore, distinct cultural beliefs and health-seeking paradigms constitute an additional layer of complexity. In these communities, traditional beliefs regarding the causes and nature of illness may pose barriers to the acceptance of and adherence to modern biomedical epilepsy care.In some instances, these traditional views may inadvertently reinforce stigma, contributing to treatment delays and perpetuating a vicious cycle: poor seizure control → psychosocial impairment → heightened stigma → further delays in seeking biomedical care → worsened seizure control.

### History of SE and patient outcomes

In contrast to the univariate analysis, a history of SE was not independently associated with anxiety after adjusting for active epilepsy and income. This suggests that the relationship between SE and anxiety may be indirect, potentially mediated through the status of seizure control: patients with a history of SE were more likely to have active epilepsy (38.9% vs. 14.5%; *P* = 0.003), which was strongly associated with anxiety.

However, despite the lack of a direct association with anxiety, SE remained independently associated with poorer quality of life. This discrepancy can be explained by several mechanisms. First, SE causes selective injury to GABAergic inhibitory interneurons in limbic structures such as the hippocampus and amygdala, leading to persistent network hyperexcitability and cognitive deficits that impair the quality of life [32]. Second, the SE-induced neuronal injury may extend beyond seizure-generating regions to involve extra-hippocampal circuitry implicated in mood regulation and cognitive processing [33, 34].

Our findings suggest that SE impacts patients’ well-being through multiple pathways extending beyond psychiatric comorbidity. Preventing SE through optimized acute management and long-term seizure control is therefore critical—not only for reducing neurological sequelae but also for preserving the quality of life.

### A path forward: integrated epilepsy and mental health care

In our study, low income and poor seizure control were found to be common risk factors for the comorbid depression, anxiety, and impairment of quality of life. This convergence underscores the need for combined epilepsy-specific and socioeconomic interventions to address the burden of psychological comorbidity and improve the quality of life in this region.

Clinically significant differences emerged in the impact patterns of the two comorbidities. Radar chart analysis revealed that anxiety was more strongly associated with persistent seizure worry—consistent with the features of generalized anxiety disorder [17]. In contrast, depression showed a weaker direct link to seizure worry but was more closely tied to the feelings of helplessness and negative expectations. This distinction suggests that psychosocial and clinical interventions must be tailored accordingly. Anxiety and depression in this population are not a homogeneous entity; they diminish quality of life through distinct psychopathological pathways. In the future, patient care should be tailored based on multi-dimensional assessment.

We therefore propose a specific pilot integrated care program in the Tibetan cultural context. This program includes: (1) improving patient adherence to antiseizure medications by providing training to village-level health workers and streamlining the drug supply chain; (2) Integrating routine mental health screening (using validated tools such as C-NDDI-E and GAD-7) into the existing epilepsy follow-up processes at town-level health centers; and (3) launching community-based stigma-reducing campaigns in collaboration with local Tibetan medicine practitioners and community leaders to address cultural misconceptions of epilepsy and mental illness.

### Limitations and future directions

This study had several limitations. First, the cross-sectional design precludes causal inference. Prospective longitudinal studies are needed to clarify the temporal relationship between SE-induced circuit-level changes and chronic psychiatric outcomes. Second, the door-to-door survey may cause underreporting due to stigma or social desirability. Third, the retrospective self-reporting may reduce the ascertainment of seizures, particularly subtle or non-convulsive ones. Fourth, 37 (22.6%) of the 164 confirmed cases were excluded from psychiatric analyses due to young age (< 16 years, *n* = 27) or severe mental disorders/intellectual disabilities (*n* = 10). This exclusion may lead to underestimation of the true psychiatric burden, as the excluded individuals may have higher neuropsychiatric morbidity. Finally, while the precision of our effect estimates suggests adequate power for primary associations, the modest single-center sample may yield unstable estimates for less common exposures and limit the generalizability. Thus, our findings should be considered as exploratory and warrant confirmation in larger, prospective multi-center studies across the Xizang plateau.

## Conclusions

Among PWE in the Tibetan area, active epilepsy is the strongest factor associated with comorbid psychiatric disorders and reduced quality of life. Socioeconomic status and disease severity also significantly impact patient prognosis. Future prospective studies are needed to establish the causality, especially to clarify whether SE-induced neurocircuitry changes directly lead to chronic psychiatric outcomes and long-term declines in quality of life. Such studies would inform targeted interventions to mitigate the enduring impact of SE on patient well-being.

## Data Availability

No datasets were generated or analysed during the current study.

## Appendix A

### Screening questionnaire as used during the large-scale survey in the community

1. Have you ever had attacks of shaking of the arms or legs that you could not control?
2. Have you ever had attacks in which you fall and become pale?

Both questions 1 and 2 must be affirmative to render the subject positive.

(3) Have you ever lost consciousness?
(4) Have you ever had attacks in which you fall with loss of consciousness?
(5) Have you ever had attacks in which you fall and bite your tongue?
(6) Have you ever had attacks in which you fall and lose control of your bladder?
(7) Have you ever had brief attacks of shaking or trembling in one arm or leg or in the face?
(8) Have you ever had attacks in which you lose contact with the surroundings and experience abnormal smells?
(9) Have you ever been told that you have or had epilepsy or epileptic fits?

Any question 3 to 9, if affirmative, renders the subject positive.

## Abbreviations

ASM: Antiseizure medcation
ANOVA: Analysis of Variance
CNY: Chinese Yuan
CT: Computed tomography
EEG: Electroencephalography
GAD-7: Generalized Anxiety Disorder-7
GDP: Gross domestic product
HPA: Hypothalamic-pituitary-adrenal
ILAE: International League Against Epilepsy
LMICs: Low- and middle-income countries
MRI: Magnetic resonance imaging
NDDI-E: Neurological Disorders Depression Inventory for Epilepsy
PWE: People with epilepsy
QOLIE-31: Quality of Life in Epilepsy Inventory-31
SD: Standard deviation
SE: Status epilepticus
TBI: Traumatic brain injury
VIF: Variance inflation factor
